# Prediction of Limb Joint Angles Based on Multi-Source Signals by GS-GRNN for Exoskeleton Wearer

**DOI:** 10.3390/s20041104

**Published:** 2020-02-18

**Authors:** Hualong Xie, Guanchao Li, Xiaofei Zhao, Fei Li

**Affiliations:** 1 Department of Mechanical Engineering and Automation, Northeastern University, Shenyang 110819, China; 1800306@stu.neu.edu.cn (G.L.); zhaoxiaofei2019@163.com (X.Z.); 2 Department of Information Science and Engineering, Shenyang University of Technology, Shenyang 110870, China; lifeisut@163.com

**Keywords:** GS-GRNN, walking on level ground, joint angle prediction, sEMG signal, plantar pressure signal, error analysis

## Abstract

To enable exoskeleton wearers to walk on level ground, estimation of lower limb movement is particularly indispensable. In fact, it allows the exoskeleton to follow the human movement in real time. In this paper, the general regression neural network optimized by golden section algorithm (GS-GRNN) is used to realize prediction of the human lower limb joint angle. The human body hip joint angle and the surface electromyographic (sEMG) signals of the thigh muscles are taken as the inputs of a neural network to predict joint angles of lower limbs. To improve the prediction accuracy in different gait phases, the plantar pressure signals are also added into the input. After that, the error between the prediction result and the actual data decreases significantly. Finally, compared with the prediction result of the BP neural network, GRNN shows splendid prediction performance for its less processing time and higher prediction accuracy.

## 1. Introduction

The issue of human–machine coordination has become one of the most important research problems in the field of robotics, especially for exoskeleton applications [1,2,3,4]. To achieve consistency and coordination of human–machine coordination, continuous prediction of the joint angle of the lower limbs is required. The common algorithms for data prediction are artificial neural network (ANN) and support vector machine (SVM). SVM is mainly used for pattern recognition with a small classification error rate, while ANN has strong ability for nonlinear mapping, self-learning and fault tolerance, which is suitable for processing inaccurate and fuzzy information.

In recent years, many scholars have studied the relevant issues. Chen Lingling proposed a natural regression algorithm based on SVM to evaluate knee joint angle and realize EMG control of lower limb prosthesis [5]. Suncheol et al. used back propagation neural network (BPNN) to predict elbow and shoulder joint angles based on upper limb EMG signals, integrated with angles calculated by dynamics, to avoid collision between the mechanical arm and the upper limbs [6]. Dai Hong et al. finished the mapping relationship between the sEMG signal of calf muscles and ankle joint angles through a GRNN network [7]. Tang et al. developed an upper-limb power-assist exoskeleton actuated by pneumatic muscles. The EMG signals from arm muscles and joint angles were collected for intention recognition to control the exoskeleton, which could be applied to assist in elbow rehabilitation after neurological injury [8]. Massimo et al. developed smart garments for lower limb motion detection in which soft sensors and readout electronics are embedded for retrieving movement. The movement of the knee and ankle joints was detected by implementing in real time a low computational algorithm, which showed high accuracy [9]. Jing Yinping et al. established a mapping relationship between sEMG signal and hip, knee and ankle joints by BPNN, and preliminarily realized the mapping control through MATLAB/ADAMS co-simulation and an off-line functional test, verifying the feasibility of the method [10].

In the previous studies mentioned above, it can be found that with the development of biotechnology, the continuous movement of the human body can be estimated by biological signals such as electroencephalo-graph (EEG) and EMG [11]; these signals are used as inputs to control the robot to follow the human body. In the process of muscle contraction, the action potential is transmitted to the skin surface and can be observed after being amplified by EMG sensors, which is called sEMG signal [12]. This signal is the potential change that occurs when the central nervous system controls muscle activity in the human body and is the summation of the action potentials of many motion units [13]. So it contains a large amount of muscle activity information and can reflect the state of muscle activity. Due to the electromechanical delay effect of muscle contraction dynamics, the generation of the sEMG signal is ahead of the generation of force and motion by about 40-100 ms [14], which is conducive to the prediction of motion intention and real-time control. Besides, sEMG can also be used to detect muscle fatigue [15]. The work presented in this paper is specialized to predict the movement of the exoskeleton wearer. Lower limb motion intention information can be got from the sEMG of the thigh muscle to realize motion pattern recognition [16], making it possible that the sEMG signal can be used as an input to the control system. In addition, this method can not only stimulate the patient’s active participation in consciousness but also encourage patients to autonomously control the muscle contraction, which is more beneficial for the recovery of motor function [17].

The previous study mentioned above has also shown that GRNN network has less processing time and higher accuracy, however, it was found that there were still large errors in the transition position during swing and support periods of predictions. The novelty of this paper lies in adding the plantar pressure signals that contain gait phase information into input data. Each joint follows a periodic trajectory in function of the gait phase. Thus, the natural repeatability could be exploited to improve the accuracy of the estimation method. So, in this paper, GS-GRNN is used to realize data prediction with the angle signals, sEMG signals and the plantar pressure signals integrated into the input data.

## 2. Data Acquisition and Processing Method

### 2.1. Overview of Data Prediction Process

**Figure 1:**
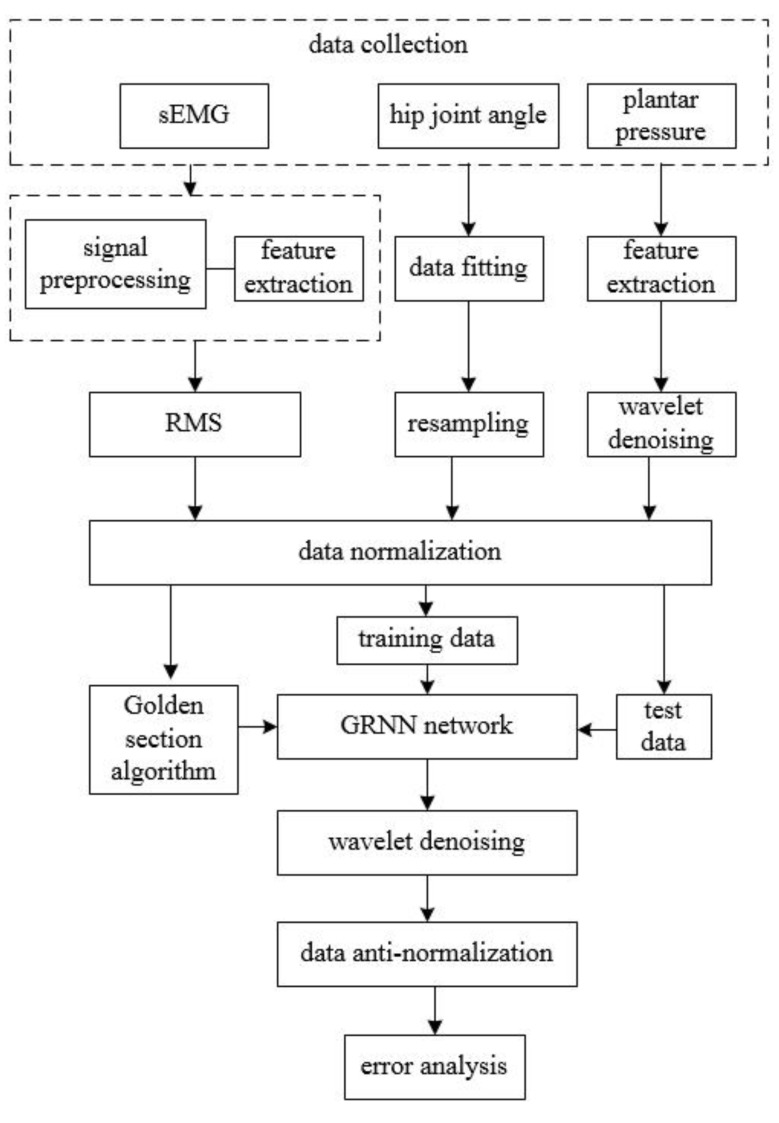
Flow chart of signal processing.

The whole system is composed of six parts, as shown in Figure 1. Part 1: The measurement of signals and the preprocessing methods are determined, such as filtering, feature extraction, data fitting, normalization, and wavelet de-noising. Part 2: The smoothing factor of GRNN is optimized based on the golden section algorithm. Part 3: The GRNN network is trained with a portion of data collected in part 1. Part 4: The rest of the data are imported into the network that has been trained in part 3 to get predicted data. Part 5: De-normalization and wavelet de-noising on the results of GRNN are performed. Part 6: Error analysis of the obtained results is made to reach a final conclusion.

### 2.2. Multi-Source Signals Acquisition Hardware

Input data in this work include lower limb joint angle signals, plantar pressure signals and sEMG signals. The hardware characteristics required for signal acquisition will be introduced next.

**Figure 2:**
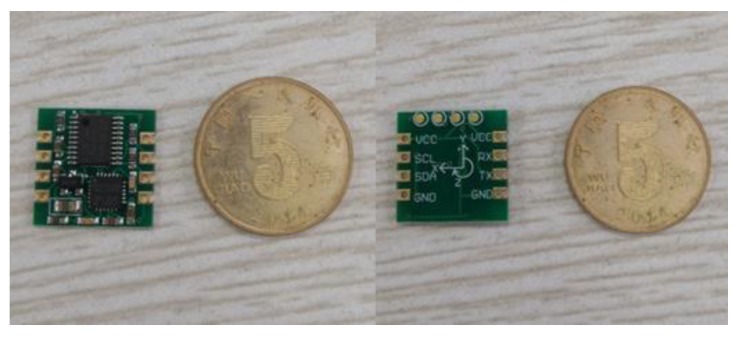
JY61 Six-axis angle sensor.

To collect lower limb joint angle signals, the JY61 six-axis angle sensor (Witmotion Shenzhen Co. Ltd., Shenzhen, China) with Kalman filtering algorithm is used in this paper, as shown in Figure 2. There are two communication modes that can be selected: serial port communication and I2C communication. In order to cooperate with the microcontroller, serial port communication is selected for this topic. The TX, RX, VCC, and GND pins corresponding to the serial communication are used to connect to the microcontroller.

**Figure 3:**
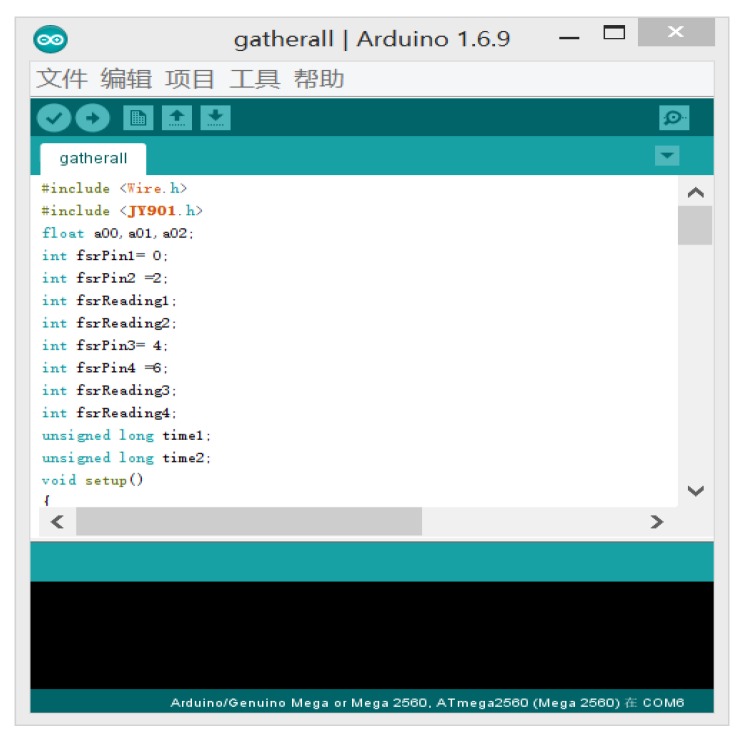
Arduino programming window.

**Figure 4:**
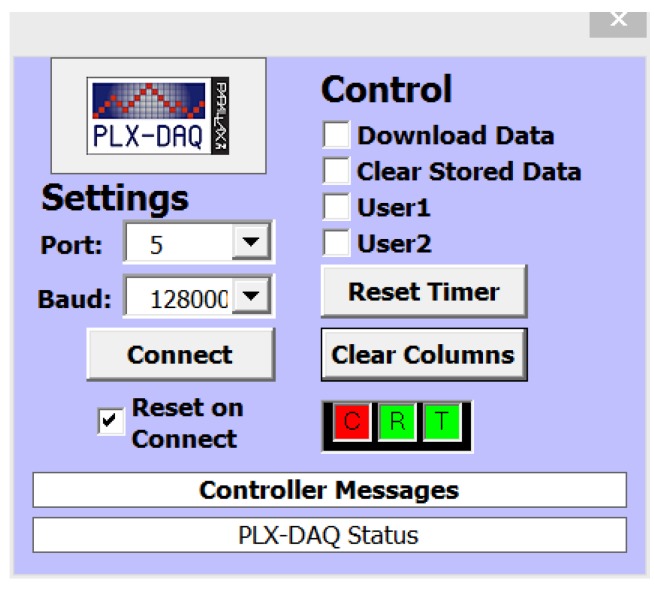
Interface of PLX-DAQ.

Three angle signals have to be collected at the same time, including hip joint, knee joint and ankle joint. The Arduino MEGA 2560 (Hesai Shenzhen Co. Ltd., Shenzhen, China) has four serial ports to meet the experiment requirements. Therefore, Arduino MEGA 2560 is used for angle signals collection. The programming interface is shown in Figure 3. In order to facilitate the later analysis of the data, PLX data acquisition (PLX-DAQ) is taken to communicate with the microprogrammed control unit (MCU) through the virtual serial port, then the angle signals are recorded into the custom excel file in real time. The interface of PLX-DAQ is shown in Figure 4.

In this paper, the selected pressure sensor and the insole are combined to make the pressure insole, so that it can measure the pressure signals from the bottom of the feet. The pressure sensor model is IMS-C20B (Aidong Wuxi Co. Ltd., Wuxi, China), which has the advantages of low price, small volume and high sensitivity. Table 1 shows the specific parameters.

**Figure 5:**
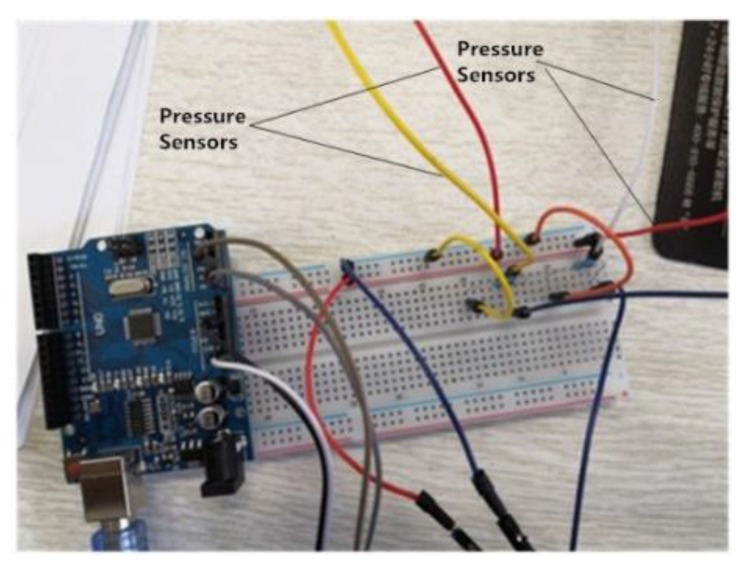
Debugging circuit of pressure sensors.

The selected pressure sensor must be connected in series with the resistance for signal acquisition. To get the appropriate resistance, debugging of the hardware circuit had to be finished on the breadboard, as shown in Figure 5.

**Figure 6:**
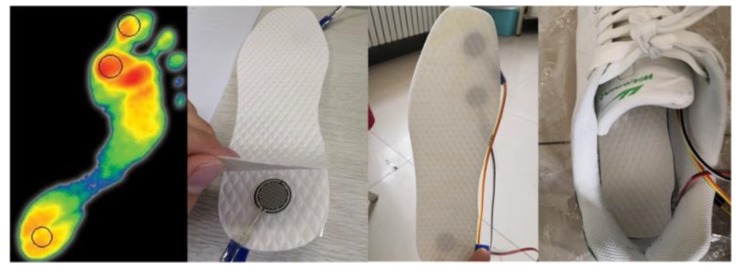
Measurement position and production of pressure insoles.

Through the analysis of numerous debugging results, the resistance of 10 kΩ was the best, under lower error and higher sensitivity conditions. Finally, the pressure sensors were connected in series with a 10 kΩ resistance and placed in the insole to make a pressure insole. In this work, the insoles were put into the sneakers that people usually wear to perform the experiment, as shown in Figure 6, so that the results are more effective.

**Figure 7:**
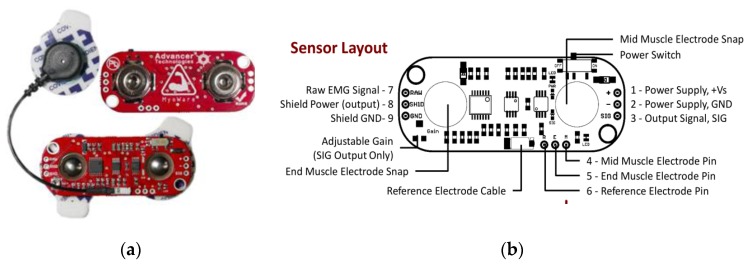
Structure of MyoWare EMG sensor. (**a**) Shape of EMG sensor; (**b**) Sensor layout [18].

**Figure 8:**
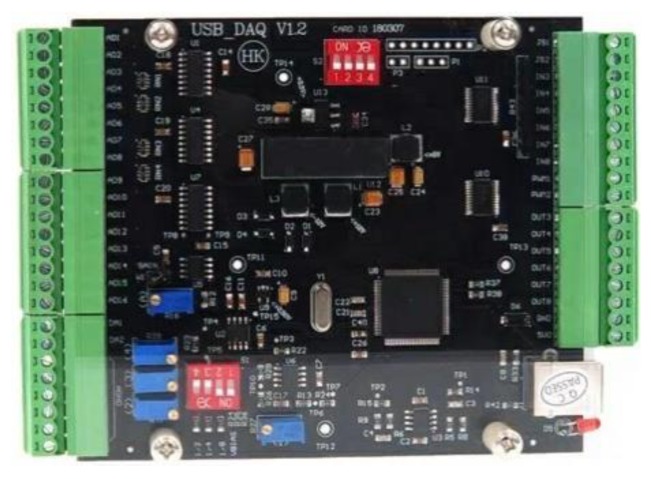
Structure of DAQ card [19].

For sEMG signals acquisition, the MyoWare EMG sensors (Advancer Technologies Littleton Company, Littleton Colorado, CO, USA) were used to measure EMG signals of thigh muscles. The structure of MyoWare EMG sensor is shown in Figure 7. Then the universal serial bus data acquisition (USB DAQ) card (HKTECH Zhengzhou Co. Ltd., Zhengzhou, China) was taken to collect the sEMG signals through analog ports. In terms of power supply, the DAQ card was powered by the personal computer (PC) through USB cable, and the EMG sensors were powered by the DAQ card. Figure 8 shows the structure of the DAQ card.

**Figure 9:**
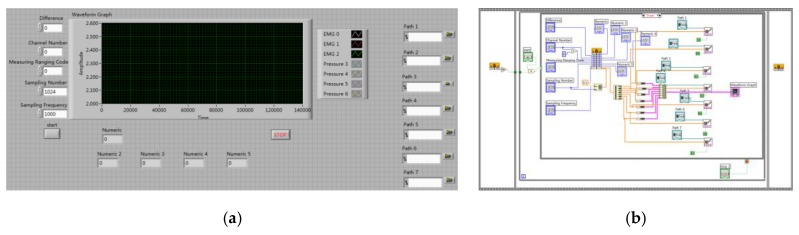
LABVIEW data acquisition program. (**a**) Front panel of program; (**b**) block diagram of program.

For data storage, LABVIEW was used to write a signal acquisition program and then data was collected in a custom file for subsequent data processing. The front panel and block diagram of LABVIEW are shown in Figure 9. The effective frequency of the EMG signal is mainly distributed in the range of 10–500 Hz; according to Nyquist’s sampling theorem, the sampling frequency must be set to at least 1000 Hz to ensure the validity of the collected signal. In this work, the sampling frequency of each channel is set to 2000 Hz.

### 2.3. Data Acquisition

Human movement of walking on level ground generally occurs in the sagittal plane, so the pitching angle during the lower limb swing process is collected as the angle signal. According to systematic anatomy, the muscles related to hip joint swing are rectus femoris (RF), biceps femoris (BF) and semitendinosus (ST), etc. Therefore, in the case of the absence of lower leg and foot, the sEMG signal of these three major muscles is collected. The input and output functions of the prediction model can be expressed as follows:

(1) y(k+1)=f(y(k),y(k−1),⋯,y(k−n+1),x(k),x(k−1),⋯,x(k−l+1)),

where y(k) is hip joint angle, and x(k) is other motion information (such as sEMG signal). The current and previous angle signal and sEMG signal are taken as inputs to estimate the joint angle at the next time.

Throughout the study, it is found that if the hip angle is only used to predict the lower limb joint angle at the next moment, the performance is poor, so the sEMG signals are taken into the input data to improve the output. However, there are still errors between the prediction results and the actual data in the transition position between the support and swing phase. It is worth considering that during the walking process, the plantar pressure also contains information about the movement of the lower limbs, which is closely related to the gait phase. Thus, the plantar pressure is added into input data, including three locations where the pressure is concentrated: The pressure at big toe, forefoot and heel. In conclusion, the input signals of the neural network include hip joint angle, sEMG signal of three major muscles, and three plantar pressure signals. 

### 2.4. Data Pre-Processing

The angle signals cannot be measured simultaneously with the EMG signals. Therefore, the angle signals and pressure signals were synchronously collected, and the EMG signals and the pressure signals were synchronously collected. Based on the number of cycles of plantar pressure signals, the angle signal and the EMG signals could be time synchronized together.

The hardware used to collect the angle signal is Arduino Mega. However, when the microcontroller communicates with the upper computer, because of the rate limit of serial port communication, the real sampling frequency is affected and the frequency is also different from that of the sEMG signals. So it is necessary to preprocess the angle signal. The processing methods are as follows: fitting the angle signal every 20 sampling points into a time curve, and then resampling the fitting results with the sampling frequency of the pre-processed sEMG signal.

The plantar pressure signal is also subjected to window processing every 20 sampling points. The average value in each window is taken as the feature value of the signal.

Because of the interference of external environmental factors such as temperature and so on, the original sEMG signal contains a lot of noise, causing a problem where the signal cannot be directly used for data prediction. Therefore, it needs be rectified and filtered to finish feature extraction. The useful EMG signal power spectrum is distributed between 30–300 Hz. The energy is mainly concentrated between 20–500 Hz. So, in this work, a Butterworth band-pass filter is designed to filter sEMG signals. Table 2 shows the major parameters of band-pass filter. There are many sEMG feature values in the time and frequency domains, such as integrated EMG (iEMG), root mean square (RMS), mean power frequency (MPF), median frequency (MF), and so on [20,21]. The RMS that can reflect the energy of the signal is chosen in this paper, which is often used to evaluate muscle activity in real time without injury [22]. The formula is as follows:

(2) $$\mathrm{RMS}=\sqrt{\frac{1}{N}\sum_{i=1}^{N}x_{i}^{2}},$$

where $x_{i}$ is the *i*th sample point of sEMG filtered by band-pass filter, and *N* is the number of the sample.

Finally, in order to improve the training and convergence speed of the neural network and reduce the impact of amplitude differences on the results, all signal data are normalized and the equation is:

(3) $$\hat{x}=\frac{x-x_{\min}}{x_{\max}-x_{\min}},$$

where x is the signal amplitude, $\hat{x}$ is the normalized amplitude of signal, and $x_{\max}$ and $x_{\min}$ are the maximum and minimum of the signal, respectively. The sEMG signal and joint angle signals are normalized into [0, 1].

### 2.5. GRNN Network Training and Prediction Results

GRNN is a kind of radial basis neural network, which has pretty good properties such as nonlinear mapping, approximation functions, quick convergence speed, and high prediction accuracy. With less sample data, the performance is also quite good. Besides, the network can also process unstable data. The Gaussian kernel function is taken as the transfer function of GRNN, and the network consists of four layers. As shown in Figure 2, there is an input layer, pattern layer, summation layer, and output layer. The input is ***X*** = [***x*_1_**, ***x*_2_**, ***x*_3_**, …, ***x*_n_**]***^T^***, and the corresponding output is ***Y*** = [***y*_1_**, ***y*_2_**, …, ***y*_k_**]***^T^***.

Suppose that the joint probability density of random variable ***x*** and ***y*** is ***f***(***x***, ***y***), and given that the observed value of ***x*** is ***X***, so the regression of ***y*** with respect to ***X***, that is, the prediction output of the neural network is:

(4) $$\hat{Y}(X)=\frac{\sum_{i=1}^{n}Y_{i}\exp[-\frac{{\left(X-X_{i}\right)}^{T}(X-X_{i})}{2\sigma^{2}}]}{\sum_{i=1}^{n}\exp[-\frac{{\left(X-X_{i}\right)}^{T}(X-X_{i})}{2\sigma^{2}}]},$$

where $\hat{Y}$ is the prediction result of ***Y*** under the condition that the input is ***X***; ***X_i_*** and ***Y_i_*** are the *i*th observation value of *x* and *y* respectively; *n* is the number of samples; *σ* is smooth factor (*σ* > 0); and $D_{i}^{2}={\left(X-X_{i}\right)}^{T}(X-X_{i})$ is the square of the Euclidean distance between ***X*** and ***X_i_***.

The transfer function of the pattern layer is:

(5) $$p_{i}=\exp[-\frac{{\left(X-X_{i}\right)}^{T}(X-X_{i})}{2\sigma^{2}}],$$

One unit in the summation layer is the denominator of Equation (1), and its transfer function is:

(6) $$S_{D}=\sum_{i=1}^{n}P_{i},$$

The other unit in the summation layer is the numerator of Equation (1), and its transfer function is:

(7) $$S_{N}=\sum_{i=1}^{n}y_{i}P_{i},$$

Thus, the result of the output layer is:

(8) $$\hat{Y}=\frac{S_{N}}{S_{D}},$$

**Figure 10:**
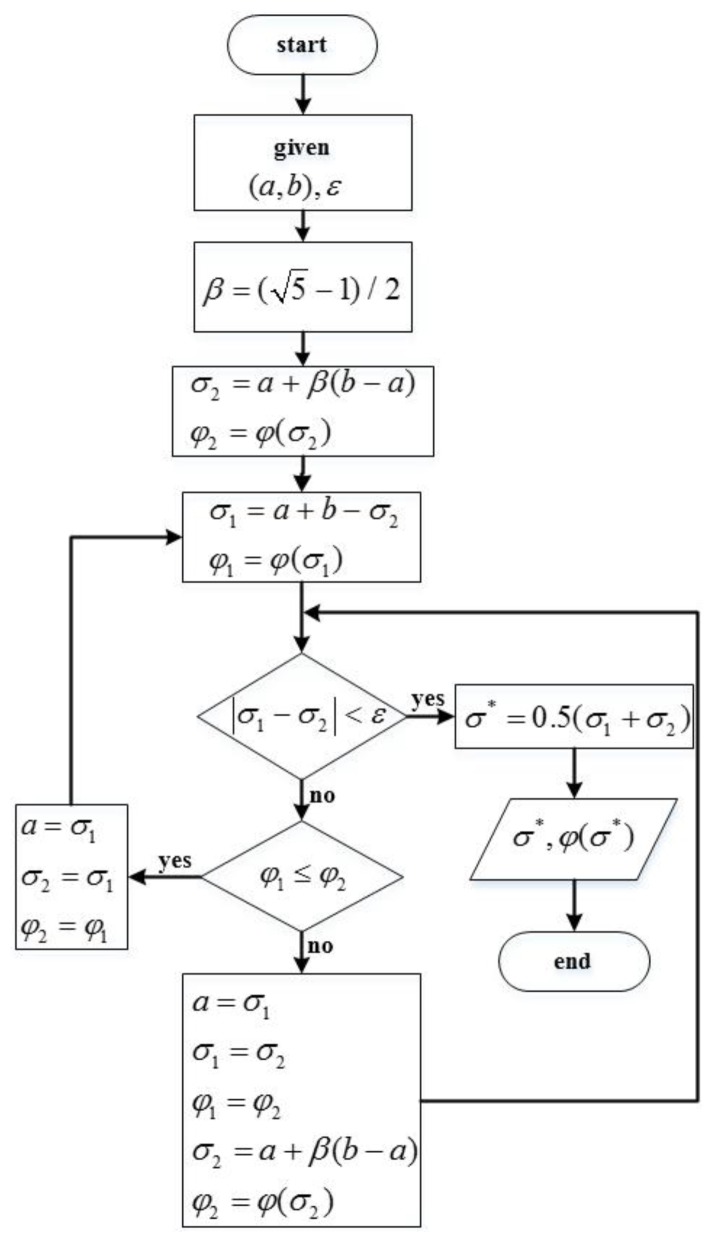
Flow chart of golden section algorithm.

The only factor that influences the performance of the network is the smoothing factor *σ*, which needs to be optimized by one-dimensional optimization method. Establishing the mean square error as the objective function between prediction results of joint angles ***Y_i_*** and actual data ***Y*** to get the optimal smoothing factor:

(9) $$E=\frac{1}{N}{\sum_{i=1}^{N}\left(Y_{i}-{\hat{Y}}_{i}\right)}^{2},$$

where *N* is the number of samples. To quickly get the optimal smooth factor, the GS algorithm is used for optimization. The algorithm, with a wide range of applications, belongs to the interval contraction method, as shown in Figure 10, where φ(t) is objective function, ε is termination, and the range of *σ* is (*a*, *b*).

**Figure 11:**
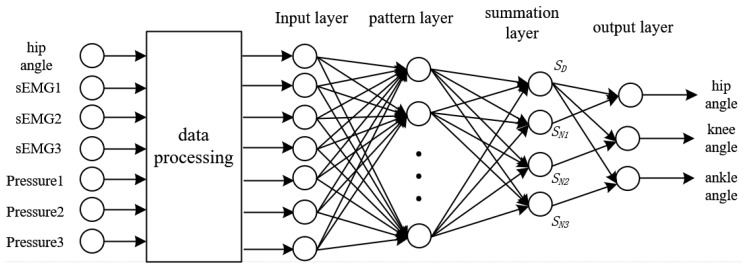
Structure of neural network.

A GRNN model is established, whose input layer contains seven neurons, including hip angle, three sEMG signals, and three plantar pressure signals; and the output layer has three neurons, including angle signal of hip, knee, and ankle joint angles, respectively. Figure 11 shows the structure of the neural network. The first two-thirds of data are used as the training set and the last third is taken as the testing set.

It was found that the prediction results of GRNN mutate within a short time, which results in a large deviation. That’s because the data becomes unstable under the influence of high-frequency signals. For the purpose of accuracy improvement, the wavelet de-noising method is adopted for signal processing by MATLAB. The basis function is coif5, and the decomposition layer number is 6. The signals are decomposed to obtain low-frequency and high-frequency signals of each layer, and then the signal reconstruction is performed by using the low-frequency wavelet coefficients obtained by decomposition so the processed signal can be obtained after removing the high-frequency noise. Finally, the signal should be de-normalized to get the predicted angle of the lower limb joint.

## 3. Experiment and Result Analysis

### 3.1. Angle Prediction Based on GRNN

**Figure 12:**
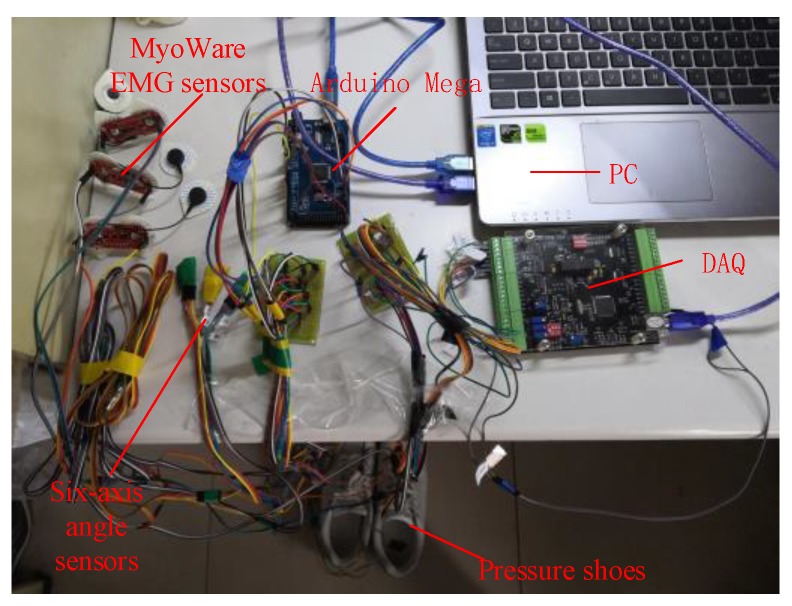
Multi-source synchronous signal acquisition system.

**Figure 13:**
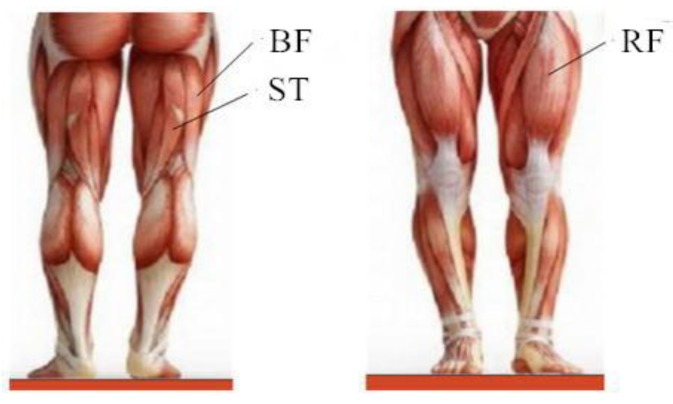
sEMG signals acquisition location [23].

**Figure 14:**
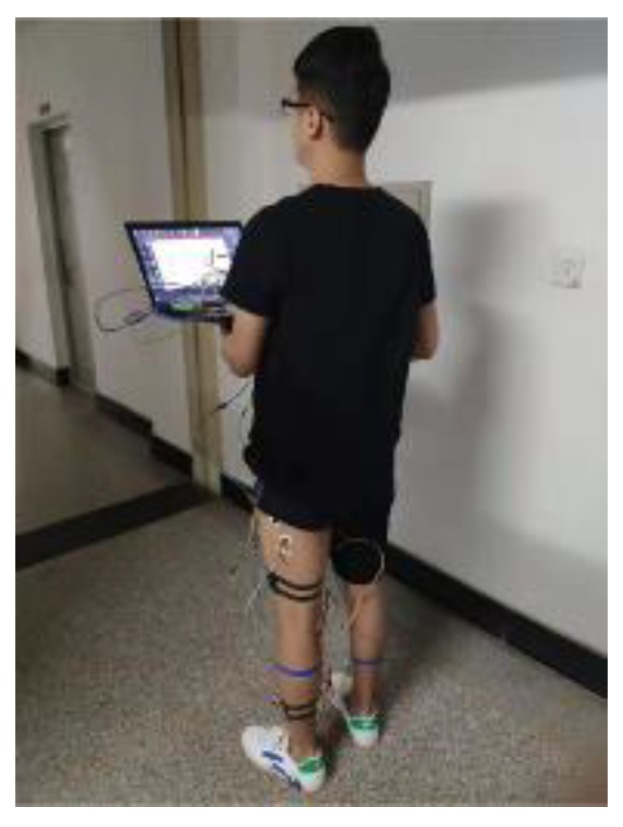
Sensor layout on the body.

The experimental subjects in this work were six healthy male students, with an average age of 25. Before the experiment, it was made sure that the experimental subjects had sufficient rest and suffered no muscle injury. In addition, the skin surface was cleaned with alcohol and then the sEMG signal, angle signal and plantar pressure signal of level walking were collected. The multi-source synchronous signal acquisition system is shown in Figure 12. Figure 13 is the sEMG signal acquisition location. Figure 14 is the sensor layout on the body.

**Figure 15:**
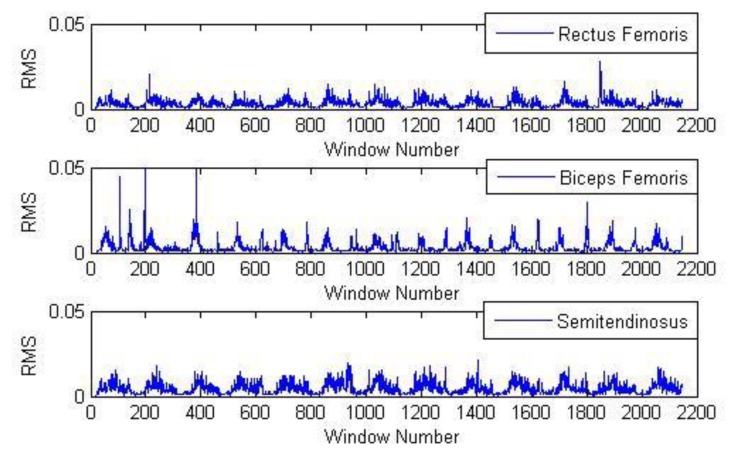
RMS feature extraction of sEMG.

From Section 2.2, the signal sampling frequency of sEMG is 2000 Hz. Since the EMG sensor was subject to the influence of temperature and other environmental factors, there was a lot of noise in the original sEMG signal. In order to retain the main information of the sEMG signal, the signals were filtered by the band-pass filter mentioned in Section 2.4. Then taking window processing for every 20 values, and calculating the RMS of each window. Taking one set of experiments as an example, Figure 15 shows the characteristic values extracted from walking sEMG signal.

**Figure 16:**
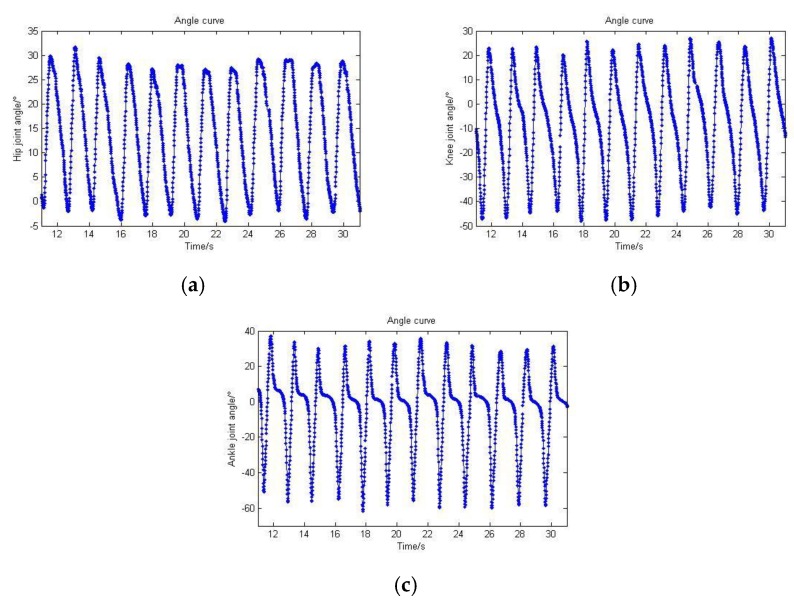
Angle data resampling while a human walks on level ground. (**a**) Hip joint angle; (**b**) Knee joint angle; (**c**) Ankle joint angle.

To remain consistent with the sampling frequency of the sEMG signal, the angle data need be fitted and then processed by resampling. The resampling frequency is 100 Hz. Results are shown in Figure 16.

**Figure 17:**
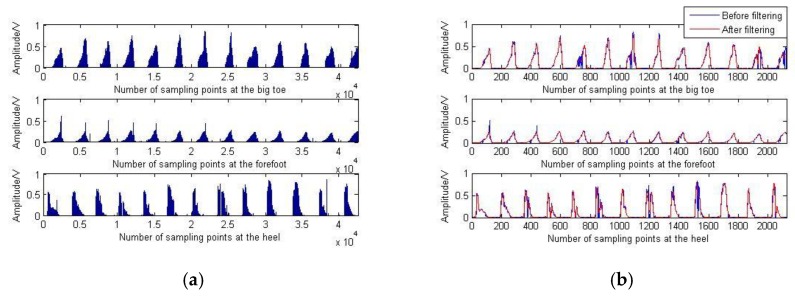
Plantar pressure signals collected during walking on level ground. (**a**) Original plantar pressure signals; (**b**) Processed plantar pressure signals.

The plantar pressure signal contains the gait phase information of the lower limb movement. As one of the input signals of the neural network, further processing is required, taking the window processing for every 20 points, as shown in Figure 17b “Before filtering”.

It can be found that there are many sudden changes in the signal, so the de-noising method mentioned in Section 2.5 is used to filter the pressure signal; the results are shown in Figure 17b “After filtering”.

**Figure 18:**
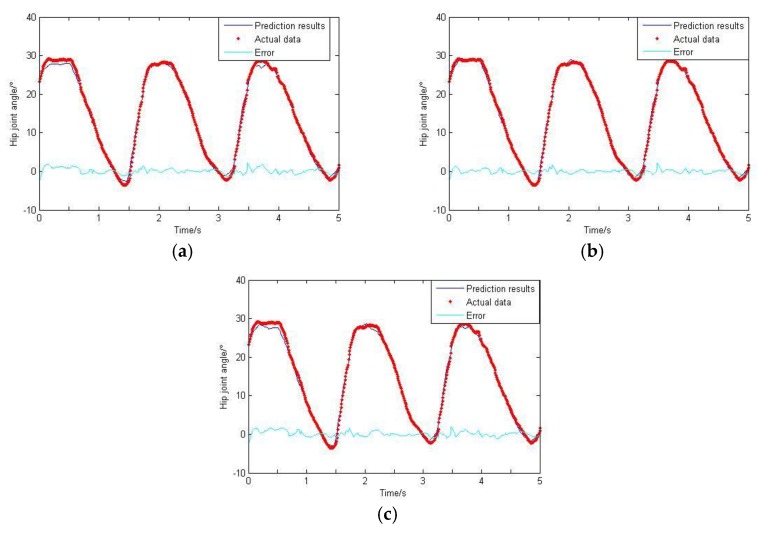
Prediction results of hip joint angle. (**a**) Taking hip joint angle as an input; (**b**) Taking hip joint angle and sEMG signals as inputs; (**c**) Taking hip joint angle, sEMG signals and plantar pressure signals as inputs.

**Figure 19:**
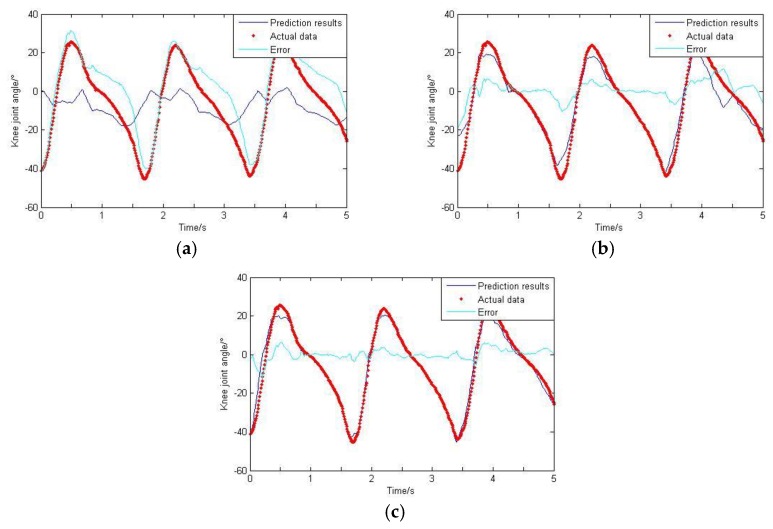
Prediction results of knee joint angle. (**a**) Taking hip joint angle as an input; (**b**) Taking hip joint angle and sEMG signals as inputs; (**c**) Taking hip joint angle, sEMG signals and plantar pressure signals as inputs.

**Figure 20:**
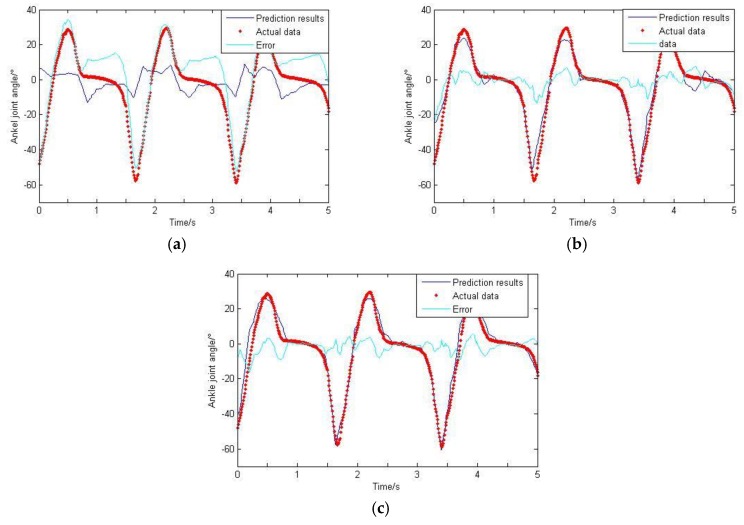
Prediction results of ankle joint angle. (**a**) Taking hip joint angle as an input; (**b**) Taking hip joint angle and sEMG signals as inputs; (**c**) Taking hip joint angle, sEMG signals and plantar pressure signals as inputs.

As can be seen from Section 2.5, the GS algorithm is adopted in this paper to get the optimal smoothing factor. The termination limit is set to 0.01, and the range of σ is (0, 0.2); the final factor is 0.023. For comparison purposes, this value is fixed. The training data is imported to train the network, and then the rest of the data is imported to test the network. The GRNN prediction results are de-noised by wavelet and carried on the de-normalization treatment. The final results are shown in Figure 18, Figure 19 and Figure 20.

### 3.2. Error Analysis

Root mean square error (RMSE) is used to estimate the deviation between the predicted value and the actual value. Mean relative error (MRE) reflects the actual change in the error between the predicted value and the actual value. In addition, correlation coefficient *γ* shows the linear correlation between predicted and actual values. The three indicators mentioned above are taken to make the error analysis.

(10) $$RMSE=\sqrt{\frac{1}{N}{\sum_{i=1}^{N}\left(x_{i}-y_{i}\right)}^{2}},$$

(11) $$MRE=\frac{1}{N}\sum_{i=1}^{N}\left|(x_{i}-y_{i})/x_{i}\right|,$$

(12) $$\gamma =\frac{\frac{1}{N}\sum_{i=1}^{N}\left(x_{i}-\bar{x}\right)(y_{i}-\bar{y})}{\sqrt{\frac{1}{N}\sum_{i=1}^{N}{\left(x_{i}-\bar{x}\right)}^{2}}\sqrt{\frac{1}{N}\sum_{i=1}^{N_{a}}{\left(y_{i}-\bar{y}\right)}^{2}}},$$

where $x_{i}$ is prediction, $y_{i}$ is actual data, *N* is number of samples, and $\bar{x}$ and $\bar{y}$ are the average value of predicted and actual measured values respectively. The final analysis results are shown in Table 3, Table 4 and Table 5.

A paper by Professor Zheng Yongping of Hong Kong Polytechnic University [24] stated that if the correlation coefficient between the predicted result and the actual measured value is greater than 0.9, and the MRE is less than 15%, then the prediction model is reliable and valid, which can be used for actual control of smart prosthesis. From this, a conclusion can be drawn that the above data prediction model is feasible.

### 3.3. Comparison with the Prediction Results of BPNN

At present, BPNN is the most widely used in engineering practice, and this neural network is generally used to realize data prediction. In this paper, the results of BPNN were taken to compare with those of GRNN. A three-layer BPNN can achieve arbitrary n-dimensional to m-dimensional mapping problems [25], so BPNN with a single hidden layer is selected in this paper. The number of iterations was 1000, the expected error was 0.001, the number of hidden layer neurons was 11, and the Levenberg-Marquardt algorithm was chosen as the training method; finally, the comparison results are shown in Table 6.

After comparison, it could be found that the calculation time of GRNN is shorter, and the correlation coefficients of the hip joint angles are almost the same. In terms of knee and ankle joint prediction, GRNN performs better.

## 4. Conclusions

Based on the GS-GRNN network, lower limb joint angle prediction is realized in this paper. When only the hip joint angle was used as an input, a low accuracy and big error were achieved. When the input included angle and sEMG signals, the accuracy and the correlation coefficient were both significantly improved. When the angle, sEMG and plantar pressure signals were simultaneously imported into GRNN together, the accuracy and the correlation coefficient were both further improved.

The innovation of this paper is to integrate the plantar pressure signal into the neural network input, which enriches the motion information of the input and improves the prediction accuracy. Therefore, the neural network is well trained and the accuracy of prediction result at each gait phase (especially the support phase) is significantly improved. Then, compared with the results of the commonly used BPNN, GRNN network is better for angle estimation of lower limbs. This work can be applied to the control of the exoskeleton in the future. Based on multi-source signals, prediction of the movement of lower limbs can enable the exoskeleton to follow the human body, which can enhance the human–machine interaction performance and improve the wearability of the exoskeleton.

## Figures and Tables

**Table 1:** Specific parameters of pressure sensors.

Model Number	Capacity	Effective Diameter	Thickness	Response Time
IMS-C20B	100 kg	20 mm	0.25 mm	<5 μs

**Table 2:** Main parameters of band-pass filter.

Parameters	Value
Pass-band Upper Cutoff Frequency (Hz)	20
Pass-band Lower Cutoff Frequency (Hz)	500
Pass-band Maximum Attenuation (dB)	3
Stop-band Upper Cutoff Frequency (Hz)	10
Stop-band Lower Cutoff Frequency (Hz)	570
Stop-band Minimum Attenuation (dB)	20

**Table 3:** Error analysis of taking angle signal as an input.

Index	Hip	Knee	Ankle
Mean Maximum Positive Error	2.9150	31.2383	34.3005
Mean Maximum Negative Error	−2.6108	−41.9477	−52.3707
RMSE	0.7538	19.3084	21.6927
MRE	0.0206	0.9062	1.4066
*γ*	0.9983	0.3583	0.3458

**Table 4:** Error analysis of taking angle signal and sEMG signals as inputs.

Index	Hip	Knee	Ankle
Mean Maximum Positive Error	3.1413	11.6854	7.5047
Mean Maximum Negative Error	−2.3674	−18.9743	−24.15
RMSE	0.5759	6.0184	6.7198
MRE	0.0145	0.0724	0.0865
*γ*	0.9987	0.9712	0.9676

**Table 5:** Error analysis of taking angle signal, sEMG signals and pressure signals as inputs.

Index	Hip	Knee	Ankle
Mean Maximum Positive Error	3.2888	6.3540	5.6428
Mean Maximum Negative Error	−2.4580	−11.6884	−16.3118
RMSE	0.7353	2.6998	3.8373
MRE	0.0194	0.0423	0.0437
*γ*	0.9985	0.9925	0.9873

**Table 6:** Comparison of BPNN and GRNN results.

Index	BPNN	GRNN
Time (s)	16.029	2.380
Hip Joint (*γ*)	0.9987	0.9985
Knee Joint (*γ*)	0.9821	0.9925
Ankle Joint (*γ*)	0.9762	0.9873
